# Development of a Routinely Applicable Imaging Protocol for Fast and Precise Middle Cerebral Artery Occlusion Assessment and Perfusion Deficit Measure in an Ovine Stroke Model: A Case Study

**DOI:** 10.3389/fneur.2019.01113

**Published:** 2019-11-14

**Authors:** Andrea Maria Herrmann, Giorgio Franco Maria Cattaneo, Sebastian Alexander Eiden, Manuela Wieser, Elias Kellner, Christoph Maurer, Jörg Haberstroh, Christoph Mülling, Wolf-Dirk Niesen, Horst Urbach, Johannes Boltze, Stephan Meckel, Mukesch Johannes Shah

**Affiliations:** ^1^Department of Neuroradiology, Faculty of Medicine, Medical Center – University of Freiburg, University of Freiburg, Freiburg, Germany; ^2^Faculty of Veterinary Medicine, Institute of Veterinary Anatomy, Histology and Embryology, Leipzig University, Leipzig, Germany; ^3^Institute for Biomedical Engineering, University of Stuttgart, Stuttgart, Germany; ^4^Department of MR Physics, Faculty of Medicine, Medical Center – University of Freiburg, University of Freiburg, Freiburg, Germany; ^5^Department of Diagnostic and Interventional Neuroradiology, University Hospital Augsburg, Augsburg, Germany; ^6^Department of Experimental Surgery, CEMT-FR, Faculty of Medicine, Medical Center – University of Freiburg, University of Freiburg, Freiburg, Germany; ^7^Department of Neurology, Faculty of Medicine, Medical Center – University of Freiburg, University of Freiburg, Freiburg, Germany; ^8^Fraunhofer Research Institution for Marine Biotechnology and Institute for Medical and Marine Biotechnology, University of Lübeck, Lübeck, Germany; ^9^School of Life Sciences, University of Warwick, Coventry, United Kingdom; ^10^Department of Neurosurgery, Faculty of Medicine, Medical Center – University of Freiburg, University of Freiburg, Freiburg, Germany

**Keywords:** CT perfusion, DSA, MCAO, reperfusion, sheep stroke model, cerebral ischemia, translational research, brain imaging

## Abstract

Temporary middle cerebral artery occlusion (MCAO) in sheep allows modeling of acute large vessel occlusion stroke and subsequent vessel recanalization. However, rapid and precise imaging-based assessment of vessel occlusion and the resulting perfusion deficit during MCAO still represents an experimental challenge. Here, we tested feasibility and suitability of a strategy for MCAO verification and perfusion deficit assessment. We also compared the extent of the initial perfusion deficit and subsequent lesion size for different MCAO durations. The rete mirabile prevents reliable vascular imaging investigation of middle cerebral artery filling status. Hence, computed tomography perfusion imaging was chosen for indirect confirmation of MCAO. Follow-up infarct size evaluation by diffusion-weighted magnetic resonance imaging revealed fluctuating results, with no apparent relationship of lesion size with MCAO at occlusion times below 4 h, potentially related to the variable collateralization of the MCA territory. This underlines the need for intra-ischemic perfusion assessment and future studies focusing on the correlation between perfusion deficit, MCAO duration, and final infarct volume. Temporary MCAO and intra-ischemic perfusion imaging nevertheless has the potential to be applied for the simulation of novel recanalization therapies, particularly those that aim for a fast reperfusion effect in combination with mechanical thrombectomy in a clinically realistic scenario.

## Introduction

Several recent randomized-controlled trials have shown that endovascular mechanical thrombectomy is highly beneficial for patients with acute ischemic stroke and large vessel occlusion (LVO) (1). This breakthrough in acute stroke treatment has led to steadily increasing numbers of patients undergoing endovascular treatment with recanalization, providing options for novel combined treatment strategies. For instance, companion neuroprotective therapies are believed to augment the beneficial impact of recanalization therapies in future settings (2, 3).

Although ischemia/reperfusion rodent models exist, these models have limitations in simulating endovascular approaches under conditions which are similar to a clinical intervention in humans. The major limitation are the much smaller vessels which, for instance, would not allow to test intravascular test devices used for or to support thrombectomy. Large animal models can fill this gap by providing a suitable vascular anatomy and size for preclinical evaluation of new endovascular or combination treatment concepts for LVO stroke (4). Non-human primate and canine stroke models are restricted by ethical concerns and high mortality in the acute and subacute stages after stroke, preventing long-term assessment of functional outcome and final lesion size as the most important clinical endpoints. Alternatively applied porcine and ovine models are more suitable to monitor long-term impact of an intervention, but exhibit a rete mirabile which does not allow direct endovascular access to the middle cerebral artery (MCA) for occlusion (MCAO). Stroke models using these species therefore require surgical access to the MCA. Recently, ovine permanent and transient MCAO stroke models were established (5, 6). Effective occlusion of the MCA main trunk or its branches was reported to depend on the qualitative visual assessment of the operating surgeon, but this may only be predictive in permanent occlusion studies. In reperfusion studies, the individual extent and capacity of collaterals can cause significant variations in final lesion volume similarly to the situation in human LVO stroke. Thus, a reliable, rapid and unbiased estimation of the perfusion deficit during MCAO is an important prerequisite for acute and long-term MCA recanalization studies. Investigating how the initial diffusion deficit corresponds to final infarct size is another important aspect awaiting clarification.

In this feasibility study, we tested several imaging modalities for application in acute ovine MCAO modeling human LVO stroke. We specifically aimed to assess (i) the reliability to confirm successful transient MCAO, (ii) MCA territory hypoperfusion, and (iii) feasibility of the imaging strategy in an experimental MCAO setting only offering a short imaging time window between vessel occlusion and reopening. This work is also intended to report pitfalls and challenges we faced during this development. We finally want to share the experience we have gained with other groups in the field, or trying to access it.

## Materials and Methods

### Animal Baseline Data

The study involved 10 merino sheep half breed (age, 1–3 years; weight, 80.2 ± 7.4 kg), kept in the CEMT-FR (Center for Experimental Models and Transgenic Service, Freiburg, Germany) under following conditions: straw boxes, daily grazing, water and hay *ad libitum*, concentrated feed pellets as reward and to foster human familiarization.

### Anesthesia

Anesthesia was prepared by intramuscular injection of midazolam [0.5 mg/kg bodyweight (BW)] and ketamine hydrochloride (20 mg/kg BW), and was induced by intravenous propofol administration (2–4 mg/kg BW). Following endotracheal intubation, 12–15 breaths/min were provided by a volume-controlled ventilator at a 10–15 mL/kg BW tidal volume and 5-mbar positive end-expiratory pressure. Settings were adjusted to normalize oxygen and carbon dioxide tension, and pH values.

Anesthesia for surgical and endovascular procedures was maintained by isoflurane in oxygen/air (FiO_2_ > 0.4), intravenous ketamine (10 mg/kg BW/h) and fentanyl (2–3 μg/kg BW/h) administration. For CT perfusion and CT angiography as well as brain MRI and angiography anesthesia was maintained by intravenous propofol (15–18 mg/kg/h).

Fluid homeostasis was maintained by intravenous infusion of Ringer solution (10 mg/kg BW/h). Electrocardiogram and blood pressure were monitored continuously. A postsurgical antibiotic (dihydrostreptomycin sulfate 12.9 mg/kg, benzylpenicillin-procaine 8 mg/kg) and analgesic (carprofen 4 mg/kg) treatment was performed.

### Surgical MCA Preparation, Occlusion, and Recanalization

Sheep were placed in the supine position slightly elevating the right shoulder. The head was then tilted to the left by ninety degrees. The wool between the ear and eye was shorn, and sterile draping was applied to cover the surgical field.

**Figure 1:**
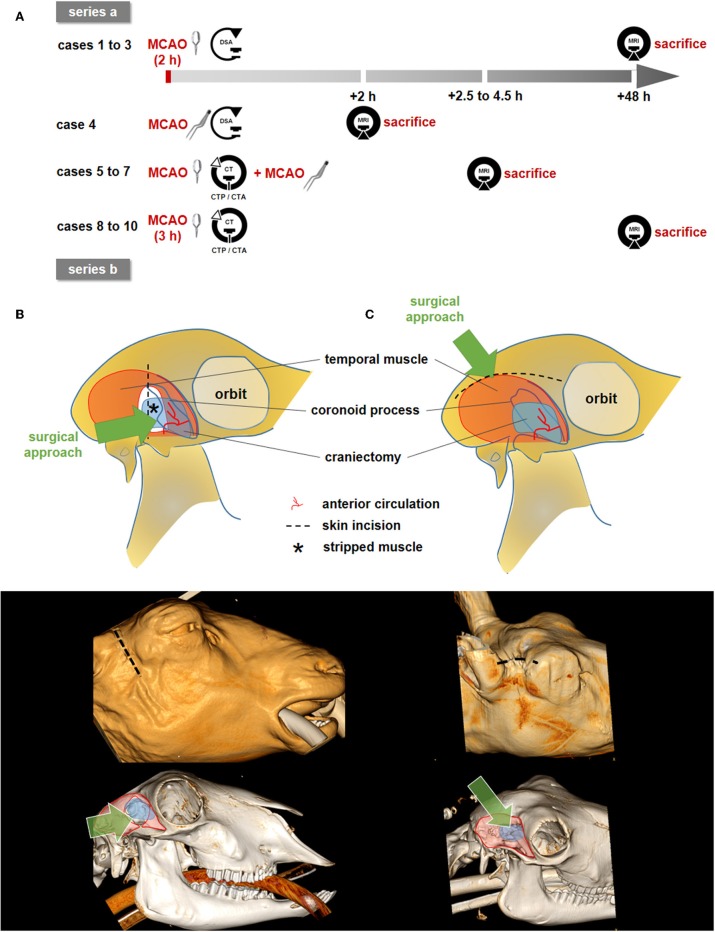
Study design and surgical approaches. **(A)** Overview on study design with two experimental series. Clip pictograms indicate (transient) MCAO by vessel clipping whereas the forceps indicate MCAO by electrocoagulation. **(B,C)**: Schematic illustration of the surgical approaches (top), 3D-Reconstruction showing the skin incision (middle), 3D-bony-reconstruction with muscle (red), and craniectomy (blue) overlays (bottom). **(B)** The surgical approach according to Wells et al. (series a). The proximal MCA and terminal ICA were reached easily. Bony CT reconstruction shows the partial removal of the coronoid process. **(C)** Approach according to Boltze et al. (series b), in which the distal branches of the MCA were followed proximally until the optic nerve and the terminal internal carotid artery (ICA) had been identified. A partial resection of the coronoid process was not necessary (CT reconstruction). Dotted lines: skin incision; blue areas: craniectomy; *: muscle dissection (series a only). The green arrows describes the surgeon's approach and the approximate line of vision.

Two different approaches to the MCA were performed. MCAO surgery in the first series of experiments (series a, cases 1–3) was carried out as described by Wells et al. (6), with the following modifications (Figure 1B). A 5 cm vertical incision was made, terminating at the zygomatic arch. Temporal and other mastication muscles were divided and stripped from the coronoid process of the mandible. Partial removal of the coronoid process was omitted when accessing the proximal MCA. The remaining masticators were then divided and stripped from the outer table as far rostral as the fibrous ring attaching the posterior orbit to the concave border of the parietal bone. Thereafter, a small craniectomy over the junction of the parietal and squamous temporal bones was performed using an electric high-speed drill (microspeed, Aesculap, Tuttlingen, Germany) to access the floor of the middle cranial fossa directly behind the orbita. The dura was then opened carefully. A 3 Head VM-900 surgical microscope (Möller-Wedel, Wedel, Germany) was used for surgical preparation of the proximal MCA and terminal ICA. The proximal MCA was occluded by a Yasargil temporary titanium clip (Aesculap) for 2 h.

Surgery in the second series (series b, cases 4–10) was carried out as described by Boltze et al. (5) (Figure 1C). The skin between the eye and ear was incised at 5–7 cm along the superior temporal fossa. The fascia of the temporal muscle was opened and the muscle was stripped away in lateral manner to expose the temporal fossa. During this maneuver, the coronoid process was lateralized and thereafter kept laterally with a self-holding spreader. The remaining masticators were then stripped from the outer table of the cranium as far rostral as the fibrous ring attaching the posterior orbit to the concave border of the parietal bone.

Craniectomy was performed as described for series a. The distal branches of the MCA were followed proximally until the optic nerve and the terminal internal carotid artery (ICA) had been identified. The MCA was permanently occluded using an electrocoagulation device (KLS Martin, Mühlheim, Germany) in case 4. This was performed to control for the influence of the exact occlusion site. In cases 5–7, a clip was placed on the MCA and left in place during CT imaging. The clip was then removed and the vessel was immediately electrocoagulated at the same location (Figure 1A). MCAO varied between 2.5 and 4.5 h depending on the particular research question to be addressed in each case. In cases 8–10, the clip was placed on the MCA and removed after 3.0 h without subsequent electrocoagulation (Figure 1A).

### Endovascular Procedure

MCAO was immediately followed by surgical cut down of the femoral artery for introduction of a 12F sheath by an experienced veterinarian (J.H.). An 8F 90-cm sheath (Flexor Shuttle Guiding Sheath; Cook, Bloomington, Indiana, USA) was then inserted into the right common carotid artery (CCA) using a coaxial 125 cm 5F vertebral or Simmons-2 shaped inner catheter for vessel selection by an experienced interventional neuroradiologist (S.M., C.M.). Selective digital subtraction angiography (DSA) with injections of contrast media (Solutrast 300, Bracco Imaging Deutschland, Konstanz, Germany) into the right CCA that was performed using a C-arm monoplanar angiography system (XA BV300, Philips Health Systems, Hamburg, Germany). Angiographic imaging for visualization of the clip-occluded right MCA was performed in variable angulations.

### Brain MRI and MR Angiography

Magnetic resonance imaging (MRI) was performed on a 3T MRI Scanner (Trio, Siemens, Erlangen Germany) using a combined 12-channel head/neck coil. The MRI protocol included sequences as shown in Table 1.

**Table 1:** Summary of 3 Tesla MRI sequence parameters.

**MRI sequence**	**Sequence parameters**	**Orientation**	**Voxel size**	**Acquisition time**
3D FLAIR	TE/TR, 395 ms/5,000 ms; TI, 1,800 ms; FA, 15°; NA, 1; IPAT, 2	sagittal	1.0 × 1.0 × 1.0 mm	5.52 min
3D MPRAGE	TE/TR, 2.15 ms/1,400 ms; FA, 15°; NA, 1; IPAT, 2	sagittal	1.0 × 1.0 × 1.0 mm	3:27 min
TSE T2	TE/TR, 95 ms/4,090 ms; FA, 140°; IPAT, 2; NA, 1	axial	0.4 × 0.4 × 0.4 mm	2:29 min
TSE T2	TE/TR, 102 ms/5,660 ms; FA, 140°; IPAT 2; NA, 1	sagittal	0.7 × 0.7 × 3.0 mm	2:23 min
TSE T2	TE/TR, 95 ms/4,911 ms; FA, 140°; NA, 1	coronal	0.4 × 0.4 × 3.0 mm	4:51 min
3D TOF MRA	TE/TR, 3.85 ms/23 ms; FA, 18°; 3D slabs, 3; NA, 2; IPAT, 2	coronal	0.5 × 0.4 × 0.6 mm	11:14 min
DWI	TE/TR, 87 ms/4,700 ms; NA, 3; IPAT, 2	coronal	1.3 × 1.3 × 3.0 mm	1:12 min
DWI	TE/TR, 86 ms/3500 ms; NA, 3; IPAT, 2	axial	1.3 × 1.3 × 3.0 mm	1:12 min

*DWI, diffusion weighted imaging; FA, flip angle; FLAIR, Fluid-attenuated inversion recovery; IPAT, integrated parallel imaging techniques; MPRAGE, Magnetization Prepared Rapid Acquisition GRE (gradient echo); NA, number of averages; TE, echo time; TOF MRA, time-of-flight MR angiography; TR, repetition time; TSE, turbo spin echo*.

Volumetric analysis of ischemic volume (on coronal DWI) and volume of edema (ischemic area plus surrounding edema on coronal T2w) was based on manual segmentations using the medical imaging platform NORA (http://www.nora-imaging.org). Ischemic areas were classified as such after correlation with generated ADC maps. Image evaluation and infarct localization was performed by an experienced neuroradiologist (S.M., C.M.) on a PACS station.

### CT Perfusion and CT Angiography

Cases 5 to 10 in series b were transferred to a 16-slice computed tomography (CT) scanner (Somatom Sensation 16, Siemens) immediately after surgical clip placement. Plain CT of the brain was performed in coronary plane sequential acquisition (5-mm slice thickness) to localize the surgical clip and to rule out intracranial hemorrhage. Then, a CT perfusion (CTP) scan was performed covering a 2.4 cm slab of the sheep brain which was centered on the tips of the MCAO clip within the MCA territory (four slices; 6-mm slice thickness). Post-processing of standard perfusion maps (CBV, CBF, and Tmax) was conducted using a dedicated commercial software package (SyngoVia, Siemens). These perfusion maps were rated by an experienced neuroradiologist (S.M.) for presence and degree of MCA territory hypoperfusion using the following semiquantitative score: 0 = no lesion visible on Tmax/CBF/CBV, 1 = lesion visible on Tmax only, 2 = lesion visible on Tmax and partially visible on CBF/CBV, 3 = lesion visible on Tmax/CBF and partially on CBV. Finally, thin-section CT angiography of the craniocervical arterial vasculature (slice thickness; 0.75 mm) was performed with arterial bolus tracking. Assessment of CTA 3D datasets was conducted by an experienced neuroradiologist (S.M., C.M.) on a PACS station.

### End of Experiments

Sheep were killed in deep anesthesia by an intravenous potassium chloride overdose at the end of each experiment (after MRI acquisition on day 2 in cases 1–3 and 8–10 and on day 0 in cases 4–7). Death by cardiac arrest was certified by an independent veterinarian.

## Results

All procedures were performed without major complications. No sheep suffered from any clinical complications except for neurological deficits after MCAO. Physiological parameters were continuously monitored before and directly after MCAO, and were in normal ranges throughout the experiments. Mean arterial blood pressure (MAP, median [IQR]) was 93 [82.25–103.75] mmHg/92 [82–107] mmHg, and pulse rate was 78 [71–99] beats/min/81 [73–97] beats/min before/after MCAO, respectively. Both parameters did not differ significantly between pre- and intraischemic measurements (*p* = 0.903 for MAP and *p* = 0.451 for pulse rate). Imaging results from all experiments are summarized in Table 2.

**Table 2:** Summary of MCAO technique, MRI findings, and visualization of MCAO with various imaging modalities.

**Animal No**.	**MCAO**		**MRI findings**				**Visibility of MCAO on vascular imaging modalities**			
	**Surgical technique**	Duration of ischemia^*^ [hours]	**Day of MRI**	**DWI lesion volume [ml]**	**T2 lesion volume [ml]**	**Location of ischemia/surrounding edema**	**TOF MRA^**#**^**	**CT Angiography**	**CT Perfusion –hypoperfusion score^§^**	**DSA of CCA/rete mirabile**
1	Clip	2	2	1.7	4.8	Midbrain**/**No	NA	NA	NA	NA**/**NA
2	Clip	2	2	25.6	17.0	Large MCA infarct**/**No	NA	NA	NA	NV**/**NA
3	Clip	2	2	0.5	3.6	Small MCA infarct**/**Yes	NA	NA	NA	NV**/**NA
4	Coagulation	2	0	13.3	5.8	Large MCA ischemia**/**No	Visible	NA	NA	NV**/**NV
5	Clip for CTP - coagulation for MRI	2.5	0	2.2	0	Medium MCA ischemia**/**No	Visible	NV	1	NA**/**NA
6	Clip for CTP - coagulation for MRI	4.5	0	14.5	11.3	Large MCA ischemia**/**No	Visible	NV	NV - Artifacts	NA**/**NA
7	Clip for CTP - coagulation for MRI	4.0	0	16.8	0.2	Large MCA ischemia**/**No	Visible	NV	3	NV**/**NA
8	Clip	3.0	2	6.0	5.4	Medium MCA ischemia**/**No	NA	NV	1	NV**/**NA
9	Clip	3.0	2	8.5	9.2	Medium MCA ischemia**/**minimal	NA	NV	3	NV**/**NA
10	Clip	3.0	2	0.9	3.9	Small MCA infarct**/**Yes	NA	NV	2 - clip Artifacts	Partially visible**/**NA

* *refers to time interval from start of MCAO until MRI DWI was performed in cases 4–7 (vessel coagulation)*.

§ *refers to semiquantitative visual assessment of hypoperfusion in MCA territory: 0, no lesion on Tmax/CBF/CBV; 1, lesion visible on Tmax only; 2, lesion visible on Tmax and partially visible on CBF/CBV; 3, lesion fully visible on Tmax/CBF and partially on CBV*.

*NV, not visible; NA, not available*.

### Results From Series a

#### Case 1

**Figure 2:**
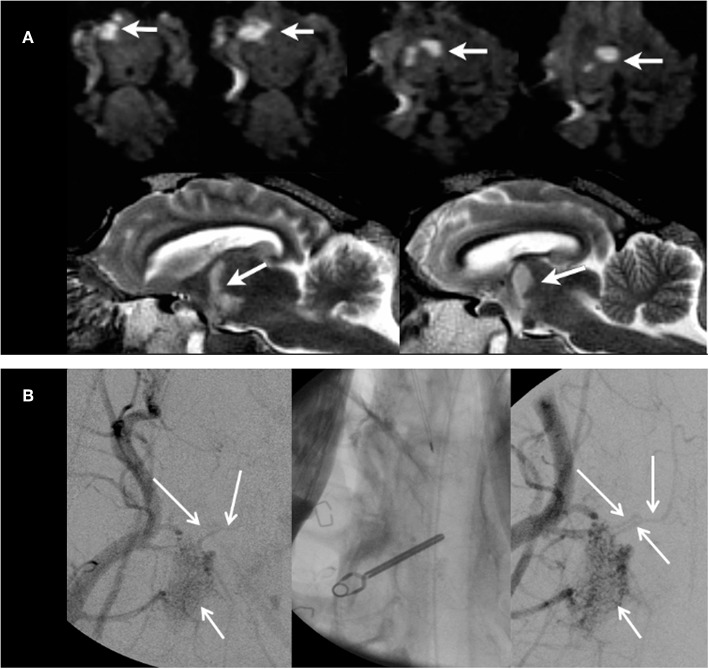
Results from case 1. **(A)** MRI images on day 2 after MCAO from case 1. Upper panels show DWI images in coronal view with ischemic lesions of the midbrain tegmental area within the right crus cerebri and right thalamus (white arrows). Lower panels show consecutive edema on T2w images in mid-sagittal views. **(B)** CCA DSA images before and after clip MCAO. After clip MCAO, no clear cut-off of MCA main trunk was visible with possible faint MCA filling (DSA image in left panel) at the clip level (unsubtracted image in mid panel). After clip removal (DSA image in right panel), filling of the main MCA trunk was visible. However, the distal MCA branch vasculature was not seen due to vessel overlap from rete mirabile and larger extracranial arteries.

DWI and T2w MRI on day 2 after MCAO showed a small ischemic lesion (1.7 mL; Figure 2A) in right thalamic and midbrain regions after 2 h of transient clip MCAO. The midbrain ischemia suggested an erroneous confusion of the MCA main trunk (M1 segment) with the terminal ICA, resulting in occlusion of terminal ICA and thus of perforating and choroidal artery branches with mesencephalic supply. This appears likely since the proximal segment of the MCA trunk forms a steep 180° curvature with an almost parallel course to the terminal ICA at the anterior skull base of sheep (please also see case 2, Figure 2B; and case 4, **Figure 4**).

#### Case 2

**Figure 3:**
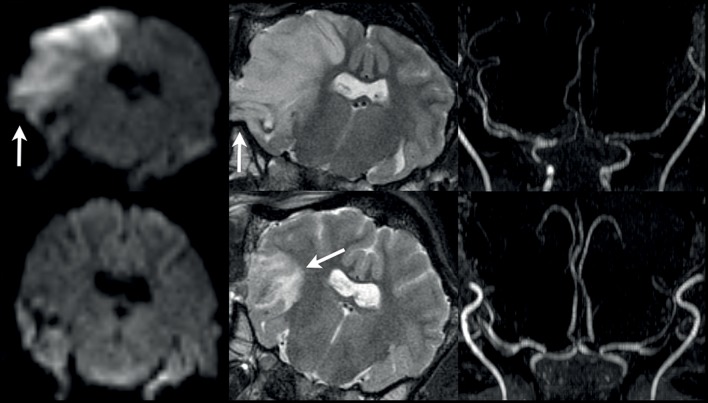
Results from cases 2 and 3. MRI images of case 2 **(upper panels)** and case 3 **(lower panels)** on day 2 after 2 h of transient MCAO. DWI and T2w images show a large right MCA territory infarct lesion (DWI lesion volume, 25.6 ml) with swelling and mild herniation through the craniectomy site (arrows in **upper left** and **mid panels**). MRA **(upper right panel)** demonstrates adequate MCA recanalization after right temporary MCAO. In case 3, no relevant MCA territory ischemia is seen on DWI images (**lower left panel**; DWI lesion volume, 0.5 ml), whereas T2w images exhibit vasogenic edema in the area of the craniectomy with mild hemorrhagic foci (arrow in **lower middle panel**). Again, MRA demonstrates adequate MCA vessel recanalization after right temporary MCAO **(lower right panel)**.

Transient clip MCAO was performed for 2 h. Selective DSA of the CCA could not unequivocally demonstrate MCA main trunk occlusion despite variable angulations of the DSA images during angiography (Figure 2B). MRI on day 2 showed a large-sized MCA territory infarct (Figure 3, upper panels) with recanalized MCA on 3D TOF MRA.

#### Case 3

Transient clip MCAO was performed for 2 h. Selective DSA of CCA during MCAO again failed to demonstrate MCA main trunk occlusion despite variable angulations of the DSA images during angiography. MRI on day 2 showed no relevant ischemia on DWI (DWI lesion volume, 0.5 ml). A small area of vasogenic edema with scattered and small hemorrhagic foci was found in the area of the surgical access to the MCAO (Figure 3, lower panels). The MCA showed a normal flow signal on 3D TOF-MRA images at day 2 after temporary clip occlusion. The neuro-deficit of the animal was light.

The chosen approach in series a (cases 1–3) resulted in a highly variable infarct configuration for two potential reasons. First, the vessel location for the surgical clip placement was inappropriate in case 1 (resulting in mid brain infarcts). Second and similar to the human situation, there might be a variable extent of MCA vessel collateral flow resulting in highly different infarct sizes between cases 2 and 3. Thus, we decided to modify the surgical approach in series b. We further tested whether the chosen MCAO location was correct by using an optimized imaging algorithm during the ischemia phase.

### Results From Series b

#### Case 4

**Figure 4:**
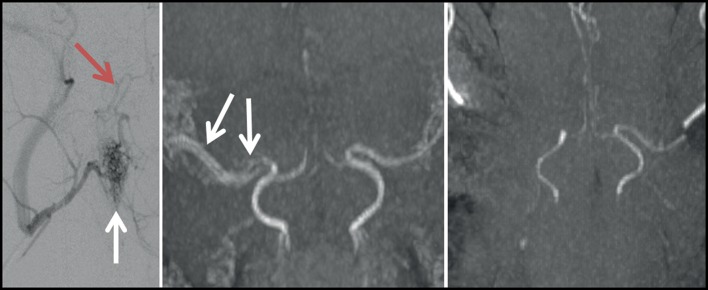
Results from case 4. DSA image **(left panel)** showing superselective rete mirabile injection (red arrow) after permanent MCAO (animal 4) without clear evidence of MCA main trunk occlusion (red arrow). 3T TOF MRA showing duplicated right MCA main trunk post temporary MCAO (**mid panel**, arrows) and clear evidence of right MCA occlusion after permanent MCAO **(right panel)**.

In this case, we tested whether DSA of the CCA with additional superselective views from injection of the right rete mirabile is capable of proofing MCAO. For immediate comparative assessment of the vessel status after MCAO on 3D TOF MRA, the MCA main trunk was electrocoagulated to avoid MRI artifacts emerging from the clip. A 0.021 inch microcatheter (Prowler Select Plus, Codman & Shurtleff, Inc., Raynham, USA) was introduced into the largest inferior arterial branch supplying the rete mirabile via long sheath endovascular access to right CCA directly after MCAO. Despite multiple angulated vessel views on superselective DSA (Figure 4, left panel), MCAO could not be correctly visualized. Further distal microcatheter navigation toward the rete mirabile led to subsequent vasospasm with impaired demonstration of downstream vasculature. MRI was performed directly at 2 h following vessel occlusion. MRA visualized the MCAO site at the MCA main trunk (Figure 4, right panel). The resulting early MCA territory infarct was visible on DWI images (DWI lesion volume 13.3 ml) with beginning edematous change on T2w images (T2 lesion volume 5.8 ml).

#### Case 5

Since DSA (including superselective views used in case 4) failed to demonstrate adequate vessel occlusion, we decided to further amend the imaging protocol by introducing CTA with CTP imaging in cases 5–7. Since electrocoagulation is not a feasible technique for transient MCAO, we decided to first perform MCAO with a clip followed by immediate transfer to CTA/CTP imaging. Thereafter, the clip was removed and the vessel was occluded in the same location by electrocoagulation in order to perform subsequent MRI without clip-borne artifacts. Thus, CTP findings could be correlated with the results of MRI simulating a temporary MCAO with ischemia duration of 2.5 h (time interval from initial vessel occlusion to MRI acquisition).

**Figure 5:**
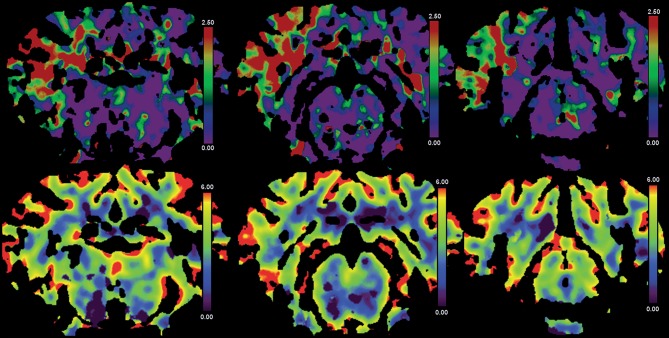
Results from case 5. CTP images from case 5. Directly after clip MCAO profound hypoperfusion is visible on Tmax maps **(upper panels)** without reduction in CBV **(lower panels)**. False color scale indicates Tmax from 0 (purple) to 2.5 s (red) in upper panels and CBV from 0 ml/100 g (purple) to 6 ml/100 g (red) in lower panels, respectively.

On CTP, a large area of right MCA territory hypoperfusion was seen on Tmax, whereas CBF and CBV maps showed no areas hypoperfusion (perfusion score 1; Figure 5). Missing flow signal of the MCA main trunk was seen on 3D TOF MRA. Visualization of the MCAO at the main trunk was not possible on CTA images due to beam hardening artifacts originating from skull bone and the clip. On DWI, signs of a small infarct in the MCA territory were detected (lesion volume 2.2 ml) without edematous change on T2w images. In this case, evidence of correct temporary MCAO at the main trunk by visualization of CTP hypoperfusion during the time window of clip occlusion was first demonstrated with good correspondence to findings in immediate MRI. Hence, duration of MCAO for 2.5 h may still have been too short to detect a fully evolved infarct.

#### Case 6

MCAO and imaging procedures were performed as described in case 5 except for the longer (4.5 h) duration of ischemia at the time of the MRI measurements in order to avoid a small final infarct due to premature recanalization. Perfusion in the right hemisphere could not be evaluated on CTP due to major streak artifacts from extensive jugular venous contrast media reflux. Correct MCA main trunk occlusion could be reliably demonstrated using 3D TOF MRA, but CTA again failed to do so due to beam hardening artifacts. Four and a half hours of ischemia led to a rather large-sized MCA infarct that was seen on DWI MRI (lesion volume 14.5 ml) with resulting early edematous changes on T2w images.

#### Case 7

MCAO was performed as described in case 5 and 6 except for a modification in the positioning of the animal during CTP acquisition in order to avoid streak artifacts originating from contrast media reflux into the jugular veins. To this end, the animal was placed in left anterior-lateral position on the CT scanner table to relieve paunch-related increase in central venous pressure. DSA was also added directly after CTP and before removal of the clip, and subsequent permanent electrocogaluation of the MCA. However, as in the previous cases, DSA images could not clearly demonstrate correct vessel occlusion. MRI was performed at 4.0 h after MCAO. MRA was able to correctly visualize MCA main trunk trunk occlusion. On CTP, a large area of MCA territory hypoperfusion was seen on and on CBF maps with hypoperfusion also visible on CBV maps (perfusion score 3). There were no major artifacts on CTP images. However, MCAO was not visible on CTA images due to beam hardening artifacts similar to cases 5 and 6. Likewise, a rather large-sized MCA territory infarct was seen on MRI (DWI volume, 16.8 ml) without significant early edematous change on T2w images after 4 h of ischemia.

#### Cases 8–10

During an interim summary of cases 5–7, the utilization of CT perfusion for demonstrating MCA territory hypoperfusion as an indicator of correct MCAO was found successful except for extensive beam hardening artifacts in case 6, caused by jugular venous reflux. Thus, we planned to gain further experience with this CT perfusion protocol (applied with modified animal positioning as described in case 7) in combination with the modified surgical approach of series b. However, we decided to continue by performing a transient clip MCAO only (omitting electrocoagulation) and infarct size measurement by MRI on day 2. The latter modifications were chosen in order to establish an imaging-based MCAO model which is designed for testing novel combined endovascular approaches of LVO stroke therapy in the future. Such transient MCAO stroke model should not only allow for ultra-early MRI but also for delayed imaging assessment of final infarct evolution and clinical follow-up as additional outcome measures.

The clip was removed after an ischemic period of 3.0 h. CTP imaging was performed directly after clip placement with modified animal positioning on the scanner table as described in case 7. Ultra-early MRI scanning was skipped and animals were allowed to wake-up and recover from the procedure. Infarct size measurement was performed on MRI at day 2 after MCAO in all three cases. On CTP, MCA territory hypoperfusion was visible on Tmax in all three cases. In addition, CBF reduction 9 and mild CBV reduction within the MCA territory was found in case 9 (perfusion score 3). In case 10, there were streak artifacts within the MCA territory from clip placement which, however, did not severely impair visibility of hypoperfusion (perfusion score 2). The MCAO was again not visible at all on DSA of the CCA in cases 8 and 9 after clip placement, and only poorly visible in case 10. On MRI at day 2, medium-sized MCA territory infarcts were evident on both DWI and T2w images (DWI volume, 6–8.5 ml) in cases 8 and 9. In contrast, the MCA territory infarction was rather small-sized (DWI volume, 0.9 ml) despite proved MCA territory hypoperfusion on CTP in case 10. This surprising result was explained by MRA on day 2 showing an early duplication of the MCA vessels as a normal variation in this case (Figure 4, mid panel). This variant may be a source for strongly improved collateralization within the MCA territory in some individuals. Identification and occlusion of the duplicate MCA main trunk can be challenging as it could be located deeply within a cerebral sulcus or the brain parenchyma.

## Discussion

The aim of this case study was to establish a feasible imaging modality for MCA territory hypoperfusion assessment in an ovine transient MCAO model, and to document our experience collected on the way toward this aim. Final infarct size on MRI represents a meaningful efficacy surrogate in experiments on acute stroke therapeutic interventions. However, in studies using transient MCAO this is only valid when the extent of brain hypoperfusion and thus the expected final lesion size without reperfusion or therapeutic intervention is known in order to compare it to the final lesion volume with recanalization and/or accompanying therapeutic intervention.

We evaluated different imaging protocols for both intra-ischemic and post-ischemic perfusion and infarct assessment, and performed a step-wise amendment of the imaging procedures and protocols. The finally resulting imaging strategy was feasible to demonstrate temporary MCA territory hypoperfusion during clip occlusion prior to vessel reopening in the intra-ischemic phase of temporary MCAO. Furthermore, we performed a “two-step” occlusion by MCA clipping prior to CTP, and electrocoagulation after CTP at the exact same vessel location to validate the results by means of TOF MRA without the risk of clip-derived artifacts.

We also determined CTP using standard post-processed image maps (Tmax, CBF, and CBV). This imaging technique was feasible to confirm hypoperfusion and thus the correct clip placement during MCAO. Such confirmation is an important quality assurance method when later removing the clip to model successful recanalization. Although derived from a relatively small number of animals undergoing CTP, our results indicate that final infarct size may be highly variable at least within a time window of 3–4.5 h of MCAO. These results are in-line with previous experiments done by Wells et al. (7) that demonstrated DWI volumes ranging from 7 to 15% of whole brain tissue after 2 h of ischemia with proximal clip MCAO in six animals. In principle, this variability may arise from incomplete MCA occlusion or a variable extent of collateral circulation to the MCA territory. Although a definite conclusion is hard to make, we argue for the latter as the most likely explanation due to numerous reasons. First, the Yasargil clips used in our experiments are also used in humans and exhibit closing forces (>150 g, 1.47 N) that should be absolutely sufficient to occlude the ovine MCA. Complete vessel coverage by the clip was confirmed after thorough visual inspection by the surgeon (M.J.S.). Of note, the ovine MCA is smaller than the vessels Yarsagil clips are usually placed on, so it is not difficult to cover it entirely. Second, we report cases of considerable infarcts (e.g., cases 2, 8, and 9). This points at a factor being different between individual subjects rather than a technical failure. Indeed, the extent of collateral circulation determines the extent of the core infarct size very early after onset of human LVO stroke of the MCA (8, 9), and the situation can be similar in sheep. If this assumption was right, it would underpin the translational value of the model described herein, but also calls for pretest assessment of collateral status to exclude extreme outcomes. In the sheep model, collateral circulation of the MCA may further be enhanced by dedicated variants of the ovine cerebral arteries such as a duplicated MCA main trunk (see case 10). Permanent MCAO by electrocoagulation as employed in our study was previously reported to result in reproducible infarct volumes throughout a 7 week surveillance period, starting 24 h after MCAO (5). Similar findings were reported for swine (10). This might come in line with our assumption, as the initially tissue-preserving effects of collateralization will become less prominent over time in case a critical hypoperfusion/complete blood flow disruption is present. During the acute stage, however, individual differences in collateralization capacity would result in much more variable lesion volumes.

The volume of hypoperfused brain tissue at early time points of vessel occlusion may be later correlated to the final lesion size. In our series, some cases that demonstrated profound hypoperfusion at the time of vessel occlusion (score 3) showed rather large-sized infarct volumes on follow-up DWI MRI. However, owing to the small number of cases with well-evaluable CTP images (*n* = 4) we were not able to clearly prove a suggested association between the extent of hypoperfusion early after clip application and final infarct on DWI by using a semiquantitative analysis of perfusion deficits. In order to provide a robust estimation of final infarcts, more data from CTP before clip removal (simulating the endovascular recanalization) should be compared to final infarct size on MRI or infarct histology in future studies.

### Surgical Approach

Reproducible and reliable infarcts could not be induced in series a, and effective occlusion depended on qualitative visual assessment by the surgeon. Due to the basal approach, the proximal MCA and terminal ICA could be reached easily. However, the narrow loop between the terminal ICA and the proximal MCA may have led to erroneous terminal ICA occlusion, resulting in brain stem infarct presumably from associated choroidal vessel occlusion with the absence of any MCA territory ischemia (case 2). Surgical knowledge of this dedicated anatomy being different to human basal brain arteries is crucial to avoid such complication. Moreover, duplication of MCA main trunk (M1 segment, see case 10 and Figure 4) represents a relatively frequently observed anatomical variant in sheep, and is also supposed to be the source for of a high degree of collateralization within the MCA territory. Such duplication may not be entirely visible during neurosurgical exposure.

According to the impression from our experienced vascular neurosurgeon (M.J.S.), the surgical approach chosen in series b was technically more suitable for MCAO, as long as possible early duplication of the MCA was ruled out and the proximal MCA (vascular loop near the optic nerve) was clearly identified.

### MCAO Imaging

DSA, superselective DSA of vessels supplying the rete mirabile, and CTA were not suitable to confirm correct MCAO. This was presumably due to the tiny caliber of intracranial arteries distal to the rete mirabile on superselective DSA and CTA, and many overlapping large-sized extracranial branches of the carotid artery on non-selective DSA, respectively. 3D rotational DSA might be an alternative option to visualize the occlusion of the MCA main trunk which was however not possible due to limited technical capabilities of our experimental angiography suite. Imaging of leptomeningeal collateral status in MCAO may be of interest as it could serve as an estimate for clinical outcome and final infarct size. However, the same methodological limitations of DSA and CTA as in the confirmation of correct MCAO may also account for the poor visualization of the tiny pial vessels in the sheep brain that impair a sufficient analysis of collaterals. However, in analogy to human large vessel occlusion stroke, CT perfusion penumbral imaging may also provide indirect information on the presence or absence of collaterals (11, 12). Non-human primate (NHP) models of ischemic stroke [for review see (4)] might be advantageous when assessing small-caliber intracerebral vessels and the cranial anatomy in NHPs is even closer to the human situation. However, the use of NHPs is ethically restricted in many contains, and costs related to using the species by far exceed those of other large animal stroke models. In turn, this restricts sample sizes and often severely limits endpoints that can be addressed quantitatively.

Beam-hardening metal artifacts were visible on CTA during temporary MCAO induced by titanium clip application. 3D TOF MRA at 3T reliably showed MCAO, but required (permanent) occlusion by electrocoagulation to avoid clip-borne artifacts (13). This issue might be mitigated by the use of newly developed and improved, but extremely expensive MRI-compatible clips. These are made of special titanium based alloys or Phynox, an alloy composed of cobalt, chrome, nickel, and molybdenum (e.g., Aesculap Yasargil mini clips) and cause only minimal artifacts. However, in transient MCAO, this imaging modality may be less efficient and also difficult to apply during a short ischemic time window between two neurosurgical procedures for clip placement and subsequent removal. This is particularly relevant for experiments that add additional time for endovascular procedures, e.g., for intra-arterial neuroprotective therapy, which necessitate additional navigation and placement of a catheter into the brain supplying arteries. Nevertheless, MRI-compatible clips might allow perfusion-weighted imaging sequences to assess the perfusion deficit during occlusion.

Positron emission tomography (PET) has been reported as a gold standard for experimental perfusion deficit assessment and is applicable in sheep (14, 15). Moreover, PET imaging is hardly susceptible to metal artifacts from a placed clip. However, PET imaging requires a dedicated infrastructure while its application and in particular full data analysis may be too time-consuming to be applied in acute experimental settings during a short time window of temporary vessel occlusion.

We found that CT perfusion reliably provided indirect evidence of MCAO by demonstrating MCA territory hypoperfusion already in a small number of cases. Importantly, it was the most feasible modality that could be applied in a time-efficient manner during temporary neurosurgical clip MCAO among the tested imaging techniques. In our experience, streak artifacts related to venous reflux into the internal jugular vein may be reduced by placing the animal in a left anterior-lateral position on the CT scanner table to relieve paunch-related venous pressure. This finally resulted in good diagnostic quality of the CTP images.

### Study Limitations

In this pilot study with small number of consecutive animals, no complete blinding or randomization could be performed which may potentially bias the analysis of study outcomes. However, the investigators who performed the volumetric analysis of infarct volume on MRI images were blinded to the respective animals' treatment protocols. Detailed information derived on correlation of infarct size with hypoperfusion volume could not be obtained due to variable imaging protocols and the small number of animals that finally underwent CTP, warranting additional research on this aspect. Consequently, further studies with a fixed CTP imaging protocol and ischemic time window of temporary MCAO are necessary.

## Data Availability Statement

The datasets generated and/or analyzed during the current study are available from the corresponding author on request.

## Ethics Statement

This study was carried out in accordance with the recommendations of the German animal protection law and the animal care guidelines of the European Community (2010/63/EU). The protocol was approved by the local ethics committee (Regierungspräsidium Freiburg, Germany; reference numbers #35-9185.81/G-14/85 and #39-9185.81/G-15/38). Study design is illustrated in Figure 1A. ARRIVE guidelines were followed as applicable for a pilot study.

## Author Contributions

AH: significant contribution to study design, to data acquisition, analysis of data, to article drafting, critical review of intellectual contents, and approval of final version. GC: significant contribution to study design, to article drafting, critical review of intellectual contents, and approval of final version. SE: significant contribution to data acquisition, analysis and interpretation of data, and to critical review of intellectual contents, and approval of final version. MW, EK, and CMa: significant contribution to data acquisition, analysis of data, and to critical review of intellectual contents, and approval of final version. JH: significant contribution to concept and study design, to data acquisition, and to critical review of intellectual contents, and approval of final version. CMü and HU: significant contribution to concept and study design, and to critical review of intellectual contents, and approval of final version. W-DN: significant contribution to data acquisition, and to critical review of intellectual contents, and approval of final version. JB: significant contribution to concept and study design, to data interpretation, and to article drafting, critical review of intellectual contents, and approval of final version. SM: significant contribution to experimental design image analysis, data acquisition and analysis and interpretation of data, article drafting, critical review of intellectual contents, and approval of final version. MS: significant contribution to experimental design, to article drafting, critical review of intellectual contents, and approval of final version.

### Conflict of Interest

GC was engineer at the company Acandis GmbH until August 2019. SM received fees and travel grants as member of scientific advisory board of company Acandis GmbH. The remaining authors declare that the research was conducted in the absence of any commercial or financial relationships that could be construed as a potential conflict of interest.
