# Corrigendum: Development of a Routinely Applicable Imaging Protocol for Fast and Precise Middle Cerebral Artery Occlusion Assessment and Perfusion Deficit Measure in an Ovine Stroke Model: A Case Study

**DOI:** 10.3389/fneur.2020.00046

**Published:** 2020-01-31

**Authors:** Andrea Maria Herrmann, Giorgio Franco Maria Cattaneo, Sebastian Alexander Eiden, Manuela Wieser, Elias Kellner, Christoph Maurer, Jörg Haberstroh, Christoph Mülling, Wolf-Dirk Niesen, Horst Urbach, Johannes Boltze, Stephan Meckel, Mukesch Johannes Shah

**Affiliations:** ^1^Department of Neuroradiology, Faculty of Medicine, Medical Center – University of Freiburg, University of Freiburg, Freiburg, Germany; ^2^Faculty of Veterinary Medicine, Institute of Veterinary Anatomy, Histology and Embryology, Leipzig University, Leipzig, Germany; ^3^Institute for Biomedical Engineering, University of Stuttgart, Stuttgart, Germany; ^4^Department of MR Physics, Faculty of Medicine, Medical Center – University of Freiburg, University of Freiburg, Freiburg, Germany; ^5^Department of Diagnostic and Interventional Neuroradiology, University Hospital Augsburg, Augsburg, Germany; ^6^Department of Experimental Surgery, CEMT-FR, Faculty of Medicine, Medical Center – University of Freiburg, University of Freiburg, Freiburg, Germany; ^7^Department of Neurology, Faculty of Medicine, Medical Center – University of Freiburg, University of Freiburg, Freiburg, Germany; ^8^Fraunhofer Research Institution for Marine Biotechnology and Institute for Medical and Marine Biotechnology, University of Lübeck, Lübeck, Germany; ^9^School of Life Sciences, University of Warwick, Coventry, United Kingdom; ^10^Department of Neurosurgery, Faculty of Medicine, Medical Center – University of Freiburg, University of Freiburg, Freiburg, Germany

**Keywords:** CT perfusion, DSA, MCAO, reperfusion, sheep stroke model, cerebral ischemia, translational research, brain imaging

In the published article, there was an error **in the allocation of the affiliation for the following authors: Horst Urbach and Stephan Meckel. Instead of affiliation 2 both authors should be affiliated to affiliation 1**.

The authors apologize for this error and state that this does not change the scientific conclusions of the article in any way. The original article has been updated.

